# Molecular characterisation of the *Chlamydia pecorum* plasmid from porcine, ovine, bovine, and koala strains indicates plasmid-strain co-evolution

**DOI:** 10.7717/peerj.1661

**Published:** 2016-02-04

**Authors:** Martina Jelocnik, Nathan L. Bachmann, Helena Seth-Smith, Nicholas R. Thomson, Peter Timms, Adam M. Polkinghorne

**Affiliations:** 1 Centre for Animal Health Innovation, University of the Sunshine Coast, Sippy Downs, Queensland, Australia; 2 Functional Genomics Center Zurich, University of Zurich, Zurich, Switzerland; 3 Infection Genomics, The Wellcome Trust Sanger Institute, Cambridge, United Kingdom

**Keywords:** *Chlamydia pecorum*, Molecular characterisation, Comparative analyses, Plasmid, Phylogeny, Koala

## Abstract

**Background.** Highly stable, evolutionarily conserved, small, non-integrative plasmids are commonly found in members of the *Chlamydiaceae* and, in some species, these plasmids have been strongly linked to virulence. To date, evidence for such a plasmid in *Chlamydia pecorum* has been ambiguous. In a recent comparative genomic study of porcine, ovine, bovine, and koala *C. pecorum* isolates, we identified plasmids (p*Cpec*) in a pig and three koala strains, respectively. Screening of further porcine, ovine, bovine, and koala *C. pecorum* isolates for pCpec showed that p*Cpec* is common, but not ubiquitous in *C. pecorum* from all of the infected hosts.

**Methods.** We used a combination of (i) bioinformatic mining of previously sequenced *C. pecorum* genome data sets and (ii) pCpec PCR-amplicon sequencing to characterise a further 17 novel p*Cpec*s in *C. pecorum* isolates obtained from livestock, including pigs, sheep, and cattle, as well as those from koala.

**Results and Discussion.** This analysis revealed that pCpec is conserved with all eight coding domain sequences (CDSs) present in isolates from each of the hosts studied. Sequence alignments revealed that the 21 p*Cpec*s show 99% nucleotide sequence identity, with 83 single nucleotide polymorphisms (SNPs) shown to differentiate all of the plasmids analysed in this study. SNPs were found to be mostly synonymous and were distributed evenly across all eight p*Cpec* CDSs as well as in the intergenic regions. Although conserved, analyses of the 21 p*Cpec* sequences resolved plasmids into 12 distinct genotypes, with five shared between p*Cpec*s from different isolates, and the remaining seven genotypes being unique to a single p*Cpec*. Phylogenetic analysis revealed congruency and co-evolution of p*Cpec*s with their cognate chromosome, further supporting polyphyletic origin of the koala *C. pecorum*. This study provides further understanding of the complex epidemiology of this pathogen in livestock and koala hosts and paves the way for studies to evaluate the function of this putative *C. pecorum* virulence factor.

## Introduction

Chlamydial plasmids are often referred to as virulence plasmids. They are small, highly conserved, non-integrative and non-conjugative plasmids that are not known to carry genetic cargo such as antibiotic resistance genes. Still, these enigmatic plasmids appear to be an essential part of the genome of the majority of species in the family *Chlamydiaceae*, with plasmids almost ubiquitously found in human-specific species such as *C. trachomatis* as well as in those that infect animals such as *C. psittaci*, *C. caviae*, *C. felis*, *C. muridarum*, and *C. pneumoniae* (Harris et al., 2012; Nunes & Gomes, 2014; Pickett et al., 2005; Read et al., 2013; Rockey, 2011; Thomas et al., 1997). The exceptions to the above are: (a) the related integrated plasmid in *C. suis*, which has been shown to be able to carry a tetracycline resistance (*tet*C) gene (Dugan et al., 2004); (b) *C. abortus*, which is not known to possess any plasmids (Sait et al., 2011) and; (c) very rare human naturally occurring plasmid-free *C. trachomatis* isolates (Peterson et al., 1990; Stothard et al., 1998).

An increasing number of studies have linked the chlamydial plasmid to the pathogenic potential of an infecting isolate, as well as to disease outcome. In animal models, it was demonstrated that naturally occurring plasmid-free *C. trachomatis* and/or plasmid-cured *C. muridarum* isolates were less infective and less virulent than the wild type plasmid positive ones (Russell et al., 2011; Sigar et al., 2014). Further, *C. muridarum* studies also demonstrated the critical role of the plasmid in the development and severity of intrauterine infections (Chen et al., 2015; Liu et al., 2014). The utility of the chlamydial plasmid encoded proteins has also been explored as targets for vaccine and diagnostic test development. Plasmid-cured *C. trachomatis* strains have shown potential as live attenuated vaccines against ocular chlamydial infections in primate models, by providing a complete protection against challenge by a virulent plasmid bearing strain (Kari et al., 2011). For diagnostic purposes, the plasmid secreted Pgp3 protein has been explored as a marker of chlamydial infections for both humans and animals (Donati et al., 2009; Li et al., 2008), while the *C. trachomatis* specific plasmid sequence was used as a target for commercial molecular diagnostic test for the *C. trachomatis* infections. However, the emergence of new Swedish *C. trachomatis* variants with deletion in the plasmid target sequence evaded diagnostics (Ripa & Nilsson, 2007), highlighting importance of the knowledge of chlamydial plasmid sequences and structure (Seth-Smith et al., 2009).

To date, evidence for a *C. pecorum* plasmid has been scarce. Recent ovine, bovine, and koala *C. pecorum* whole genome sequencing studies did not report the presence of a plasmid in any of the resulting genome sequences (Bachmann et al., 2014; Bachmann et al., 2015; Mojica et al., 2011; Sait et al., 2014). In our latest comparative genomics study of *C. pecorum* from a variety of hosts, we identified four complete *C. pecorum* plasmids (p*Cpec*) in the genomes of a porcine and three koala *C. pecorum* isolates, using a set of nine available previously sequenced porcine, ovine, bovine and koala *C. pecorum* genome data sets (Jelocnik et al., 2015a). Sequence analysis showed that all four p*Cpec*s were 7.5 kbp in length with eight predicted CDSs with 99% nucleotide sequence identity, and an overall nucleotide sequence identity of 67–70% to orthologous genes from chlamydial plasmids in different species (Jelocnik et al., 2015a). A subsequent PCR-based p*Cpec* screening of 114 *C. pecorum* strains from pigs, sheep and cattle, and 113 strains from koalas revealed that p*Cpec*, while present in *C. pecorum* taken from all of the infected hosts, is not ubiquitous in all *C. pecorum* isolates: p*Cpec* was detected in 38.6% of the sampled *C. pecorum* taken from livestock, while p*Cpec* was more commonly detected in the Australian koala *C. pecorum* isolates, with a 55.8% detection rate (Jelocnik et al., 2015a). This varying p*Cpec* distribution in koala and livestock *C. pecorum* strains potentially sets this plasmid apart from those described in other chlamydial species.

In the current study, we fully sequenced and characterised 17 novel p*Cpec*s from a set of 16 *C. pecorum* strains from the most common hosts of this pathogen (pigs, sheep, cattle and koalas) in order to examine the genetic structure and diversity of p*Cpec*. In doing so, we observed that, although conserved, the p*Cpec* sequences are genetically diverse, while the p*Cpec*s phylogenies indicated congruency and co-evolution with its cognate *C. pecorum* chromosome.

## Methods and Materials

### *C. pecorum* positive clinical samples and isolates used for p*Cpec* characterisation and analyses

A total of 17 novel p*Cpec* were characterised from a small collection of cultured *C. pecorum* isolates and *C. pecorum* positive clinical swab/tissue samples from two porcine, four ovine, three bovine, and seven koala hosts, previously screened positively for p*Cpec* presence. *C. pecorum* samples used in the present study and their descriptions are outlined in Table 1.

**Table 1:** Descriptions of *C. pecorum* samples used for plasmid characterisation.

Plasmid (p*Cpec*) ID	Host and country of origin	Type of sample/ Anatomical site	Host pathology	Plasmid generation	Length (bp)	GC content (%)	Accession number	Strain reference
L1^*^	Pig, Austria	Culture/Lung	Pneumonia	WGS^c^	7,548	31.7	KT223773	Koelbl (1969)
R106	Pig, Austria	Culture/Lung	Pneumonia	Amplicon seq.^d^	7,548	31.7	KT223776	Koelbl (1969)
1886	Pig, Austria	Culture/Lung	Pneumonia	Amplicon seq.^d^	7,548	31.7	KT223767	Koelbl (1969)
IPA	Sheep, USA	Culture/Joint	Polyarthritis	Amplicon seq.^d^	7,547	31.6	KT223771	Bachmann et al. (2014)
W73	Sheep, Ireland	Culture/Faeces	Asymptomatic	Amplicon seq.^d^	7,547	31.6	KT223780	Sait et al. (2014)
Cur/E19/Rec	Sheep, Australia	Swab sample/Rectum	NCD^a^	Amplicon seq.^d^	7,547	31.6	KT223769	Jelocnik et al. (2014b)
Cur/E11/Rec	Sheep, Australia	Swab sample/Rectum	NCD^a^	Amplicon seq.^d^	7,547	31.6	KT223768	Jelocnik et al. (2014b)
LW623	Cattle, USA	Culture/Brain	Encephalomyelitis	Amplicon seq.^d^	7,547	31.6	KT223774	Kaltenboeck, Kousoulas & Storz (1993)
WA/B31/Ileal	Cattle, Australia	Tissue sample/Ileum	SBE^b^	Amplicon seq.^d^	7,548	31.7	KT223781	Jelocnik et al. (2014a)
66P130	Cattle, USA	Culture/Faeces	NCD^a^	Amplicon seq.^d^	7,548	31.7	KT223766	Kaltenboeck, Kousoulas & Storz (1993)
SA/K84/Ure	Koala, Australia	Swab sample/Urethra	NCD^a^	Amplicon seq.^d^	7,547	31.6	KT223778	Jelocnik et al. (2015a)
SA/K09/Ure	Koala, Australia	Swab sample/Urethra	NCD^a^	Amplicon seq.^d^	7,547	31.6	KT223777	Jelocnik et al. (2015a)
Vic/R6/UGT	Koala, Australia	Swab sample/UGT	NCD^a^	Amplicon seq.^d^	7,547	31.6	KT223779	Jelocnik et al. (2015a)
Marsbar^*^	Koala, Australia	Culture/UGT	Cystitis	WGS^c^	7,547	31.5	KT223775	Bachmann et al. (2014)
IPTaLE^*^	Koala, Australia	Culture/Ocular	Conjunctivitis	WGS^c^	7,547	31.5	KT223772	Bachmann et al. (2014)
DBDeUG^*^	Koala, Australia	Culture/UGT	UGT infection	WGS^c^	7,547	31.5	KT223770	Bachmann et al. (2014)
HazBoEye	Koala, Australia	Culture/Ocular	Conjunctivitis	WGS^c^	7,547	31.8	KT352920	Jelocnik et al. (2015a)
HazBoUGT	Koala, Australia	Culture/UGT	Conjunctivitis	WGS^c^	7,547	31.8	KT352921	Jelocnik et al. (2015a)
NoHerEye	Koala, Australia	Culture/Ocular	Conjunctivitis	WGS^c^	7,547	31.5	KT352922	Jelocnik et al. (2015a)
TedHUre	Koala, Australia	Culture/Urethra	Cystitis	WGS^c^	7,547	31.5	KT352923	Jelocnik et al. (2015a)
PMHaUre	Koala, Australia	Culture/Urethra	Cystitis	WGS^c^	7,547	31.5	KT352924	Jelocnik et al. (2015a)

**Notes.**

* Previously characterised plasmid.

a No clinical disease.

b Sporadic bovine encephalomyelitis.

c Contig from whole genome sequencing.

d Conventional PCR overlapping fragments, dideoxy sequenced.

### Chlamydial sequences used for phylogenetic analyses in the present study

In the present study, we have also used publicly available: (i) plasmid sequences of *C. pneumoniae* pLPColN (NC017286); *C. muridarum* pMoPn (AE015926); *C. trachomatis* pCTA (CP000052); *C. avium* p10DC88 (CPOO6571); *C. felis* pCfe1 (AP006862); *C. psittaci* p6BC (CP002550); *C. caviae* pCpGP 1 (AE015926); and *C. pecorum* pCpecL1 (KT223773), and (ii) 16S rRNA gene sequences of the corresponding plasmid-bearing chlamydial strains: *C. pneumoniae* LPCoLN (FJ236984); *C. muridarum* MoPn (CP007217); *C. trachomatis* A/HAR-13 (NR025888); *C. avium* 10DC88 (NR121781); *C. felis* Fe/C56 (NC007899); *C. psittaci* 6BC (NR102492); *C. caviae* GPIC (NR036833); and *C. pecorum* L1 (LFRH01000000) for sequence and the Bayesian phylogenetic analyses.

### p*Cpec* characterisation from *C. pecorum* whole genome sequencing data

The raw Illumina MiSeq reads of five unpublished koala *C. pecorum* genomes (HazBoEye, HazBoUGT, NoHerEye, TedHUre, and PMHaUre), sequenced at Wellcome Trust Sanger Institute, Cambridge, UK, were mapped to the newly identified p*Cpec*L1, and p*Cpec*Marsbar sequences (Jelocnik et al., 2015a). Reads mapping to p*Cpec*L1 or p*Cpec*Marsbar were assembled from the data sets of five koala *C. pecorum* isolates. The resulting assemblies described a ∾7.5 kbp plasmid, each composed of a single contig (see Table 1). Assembled plasmids were aligned with the porcine p*Cpec*L1 and koala p*Cpec*Marsbar using ClustalX, as implemented in Geneious 7.1.4 (Kearse et al., 2012).

### Primers design for p*Cpec* amplicon sequencing

**Figure 1:**
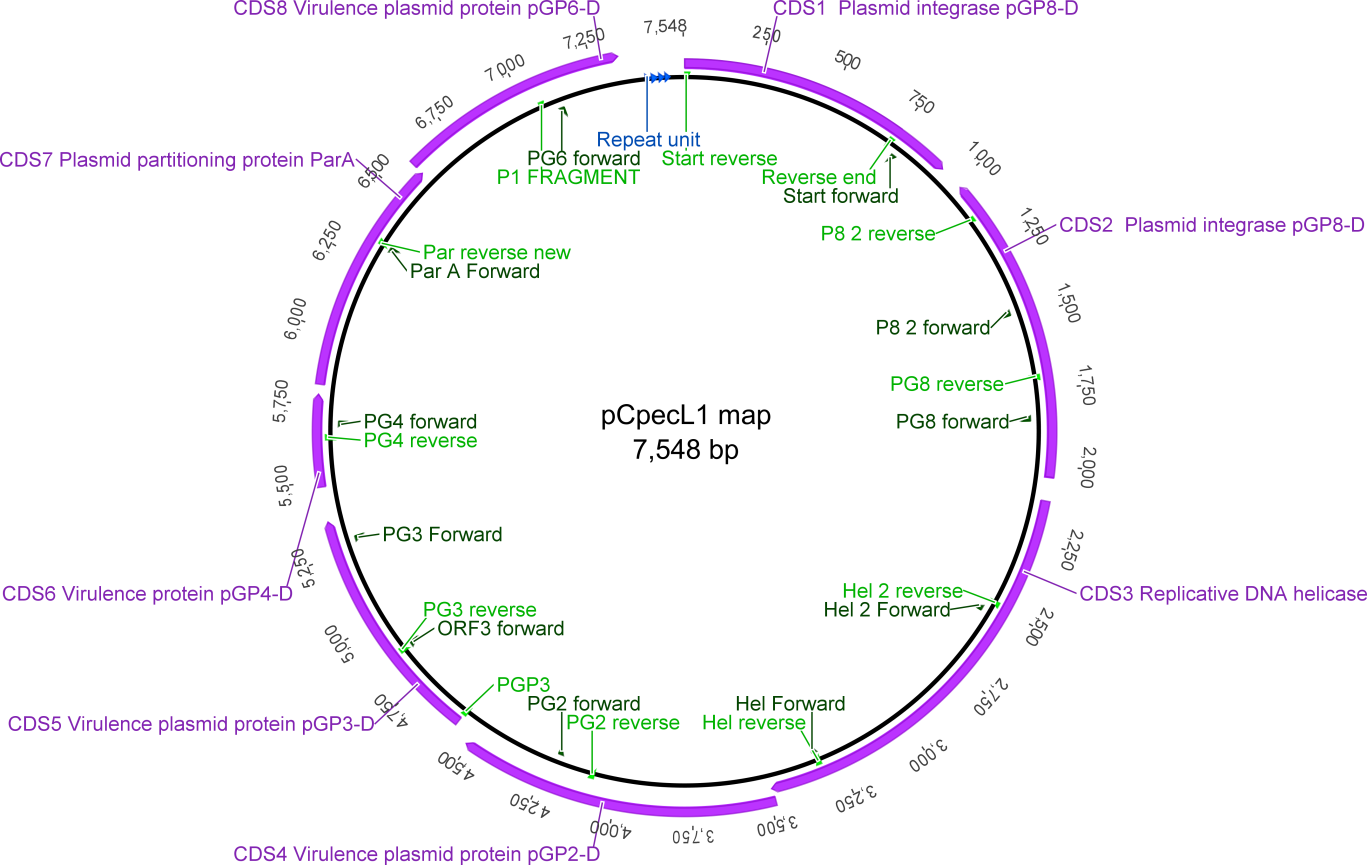
Graphical representation of the p*Cpec*L1 and annotated CDSs, including primer locations. Putative ori at the top. The 22 bp tandem repeat units are indicated by blue arrows.

Using the p*Cpec*L1 and koala p*Cpec* sequences, we designed 23 oligonucleotide PCR primers to amplify overlapping p*Cpec* fragments (Fig. 1 and Table S1). Primers were tested for DNA base mismatches using Basic Local Alignment Search Tool (BLAST) (http://blast.ncbi.nlm.nih.gov/Blast.cgi#), as well as analysed in OligoAnalyzer 3.1 online tool (https://sg.idtdna.com/calc/analyzer). Primers were designed to have similar annealing temperatures so that they could be used in various combinations to amplify products of between ∼700 bp and 3.4 kbp fragments (e.g., PG6 For and PG8 Rev: 3.4 kbp fragments; PG3 For and PGP3 Rev: 731 bp; Table S1).

A subset of 12 *C. pecorum* positive DNA samples (Table 1), obtained from various anatomical sites from porcine, ovine, bovine, and koala hosts were used for further plasmid identification and characterisation. These samples were part of the 227 sample collection previously screened for p*Cpec* presence (Jelocnik et al., 2015a), by our p*Cpec*-specific PCR. PCR amplifications for p*Cpec* fragments up to 1 kbp were performed as previously described (Jelocnik et al., 2015a), with appropriate annealing temperature (Table S1). For the amplification of fragments (>1.5 kbp) the LongRange PCR kit, Qiagen, Victoria, Australia, was used as per manufacturer instructions. Isolated p*Cpec*Marsbar, p*Cpec*IpTaLe, p*Cpec*DbDeUG DNA from koala *C. pecorum* strains MC/Mars, DBDeUG, and IpTaLE were used as positive controls. Genomic DNA from the non-plasmid containing strains L17 and L71, and dH20 were used as negative controls (Jelocnik et al., 2015a). The presence of the amplicons were confirmed on 1.5% agarose-TBE gels, and then purified and dideoxy sequenced (The Institute for Future Environments (IFE), Queensland University of Technology (QUT), Brisbane, Australia using the Applied Biosystems ABI3500 Gene analyser).

Forward and reverse chromatogram of each sequenced amplicon was aligned in Geneious 7.1.4 and the amplicon consensus sequence was extracted. Overlapping amplicon sequences were used to assemble the full length plasmid sequence for each sample. The derived p*Cpec* sequences were annotated with RAST (Aziz et al., 2008),and further curated in Geneious 7.1.4. p*Cpec*s translated CDSs were further analysed in blastp for comparison (http://blast.ncbi.nlm.nih.gov/Blast.cgi), as well as Universal Protein Resource (UniProt from http://www.uniprot.org/) and Conserved Domains Database (CDD from http://www.ncbi.nlm.nih.gov/cdd) to assess the protein functionality. Plasmid sequences were deposited in Genbank under accession numbers KT223766– KT223781, and KT352920, KT352921, KT352922, KT352923and KT352924.

### 21 p*Cpec* sequence and phylogenetic analyses

In order to assess the evolutionary relationships of p*Cpec*s and the level of sequence diversity, we determined the number of synonymous (*d_s_*) and non-synonymous (*d_n_*) substitutions found in all 21 *C. pecorum* plasmid sequences included in this study. The number of polymorphic (segregating) sites, CDS alleles, plasmid genotypes, and putative recombination events were determined using DnaSP 5.0 (Librado & Rozas, 2009). A p*Cpec* ancestral sequence was reconstructed using an alignment of all 21 p*Cpec*s performed on the FastML server (Ashkenazy et al., 2012). Best-fit models of nucleotide substitution used for phylogenetic analysis of the plasmid sequences were estimated by jModelTest v.2.1.1 (Darriba et al., 2012). A rooted Bayesian phylogenetic tree consisting of eight chlamydial plasmids or their corresponding 16SrRNA gene sequences were constructed with MrBayes as implemented in Geneious 7.1.4, with HKY + I + G for plasmid, and HKY + G for 16S rRNA sequences. Both trees used the *C. muridarum* plasmid or 16S rRNA sequences as a root. A Bayesian phylogenetic tree consisting of all 21 *C. pecorum* 7.5 kbp plasmid sequences was also constructed with MrBayes using GTR + G model. *C. pneumoniae* plasmid pLPCoLN sequence was used as an outgroup. The plasmid phylogenetic tree was compared to the chromosome Multi Locus Sequence Typing (MLST-based) phylogeny constructed from the concatenated sequence of the seven MLST house-keeping (HK) gene fragments (Jelocnik et al., 2013) for corresponding samples, where available (excluding SA/K84/Ure, SA/K09/Ure and Vic/R6/UGT ). A Bayesian MLST phylogenetic tree of 18 *C. pecorum* samples sequences was also constructed in MrBayes with the HKY85 + I + G model. Run parameters for all phylogenetic trees from this study included four Markov Chain Monte Carlo (MCMC) chains with a 1,000,000 generations, sampled every 100 generations and with the first 10,000 trees were discarded as burn-in.

## Results and Discussion

### pCpec: a newly characterised member of chlamydial plasmids

With the recent observation that the *C. pecorum* plasmid is common but not ubiquitous across this species (Jelocnik et al., 2015a), we characterised an additional 17 novel p*Cpec* sequences from *C. pecorum* strains isolated from a variety of hosts. In total, we included a set of these 21 *C. pecorum* plasmid sequences for analyses.

These data revealed that all 21 p*Cpec*s were 7.5 kbp in size with a low G + C (av. 31.6%) content, typical for chlamydial plasmids (Thomas et al., 1997). Using an alignment of eight representative plasmid sequences from related chlamydial species (including p*Cpec*), we constructed a plasmid phylogeny and compared it to the 16S rRNA gene phylogeny of the corresponding strains carrying these plasmids (Fig. S1). As observed in Fig. S1 and consistent with previous phylogenetic analysis of chlamydial plasmids from other species (Mitchell et al., 2010), plasmid phylogenies displayed a similar topology to that observed for the 16S rRNA derived chlamydial phylogeny. In this study, both *C. pecorum* p*Cpec*L1 and the *C. pecorum* 16SrRNA-gene sequence clustered with its closest chlamydial relative *C. pneumoniae* (Gupta et al., 2015). The observed phylogenetic relationships of the chlamydial plasmids also suggest that the plasmids evolve in parallel with their bacterial host (Rockey, 2011), and therefore that these plasmids were acquired early in the evolution of *Chlamydiae* and have been subject to little between-species recombination (Andersson & Kurland, 1998), although intra-species plasmid-associated recombination has been reported previously in *C. trachomatis* (Harris et al., 2012).

### pCpec phylogeny

Overall, the alignment of the 21 p*Cpec* sequences resolved 12 distinct plasmid sequence types (genotypes), with five genotypes shared between p*Cpec*s from different isolates, and the remaining seven genotypes were unique to a single p*Cpec* (Fig. S2). The three porcine p*Cpec*s were of an identical sequence type (Genotype L). Among the 11 koala p*Cpec*s, we identified five distinct genotypes, with: (i) p*Cpec*s SAK09Ure, SAK84Ure and VicR6UGT sharing the first (Genotype A); (ii) p*Cpec*s NoHerEyes, PMHaUre, TedHUre and DbDeUG sharing the second (Genotype B) and; (iii) p*Cpec*s HazBoEye and HazBoUgt sharing the third genotype (Genotype C); in contrast, the p*Cpec*s Marsbar, and IpTale were of a distinct fourth and fifth genotype each (Genotypes D and E, respectively). With the exception of the p*Cpec*s CurE11Rec, and CurE19Rec which were also of an identical sequence type (Genotype F), the remaining two ovine (W73 and IPA), and the three bovine (WAB31Ileal, 66P130, and LW623) p*Cpec*s were of a unique genotype each (Genotypes G, H, I, J and K, respectively) (Fig. S2).

**Figure 2:**
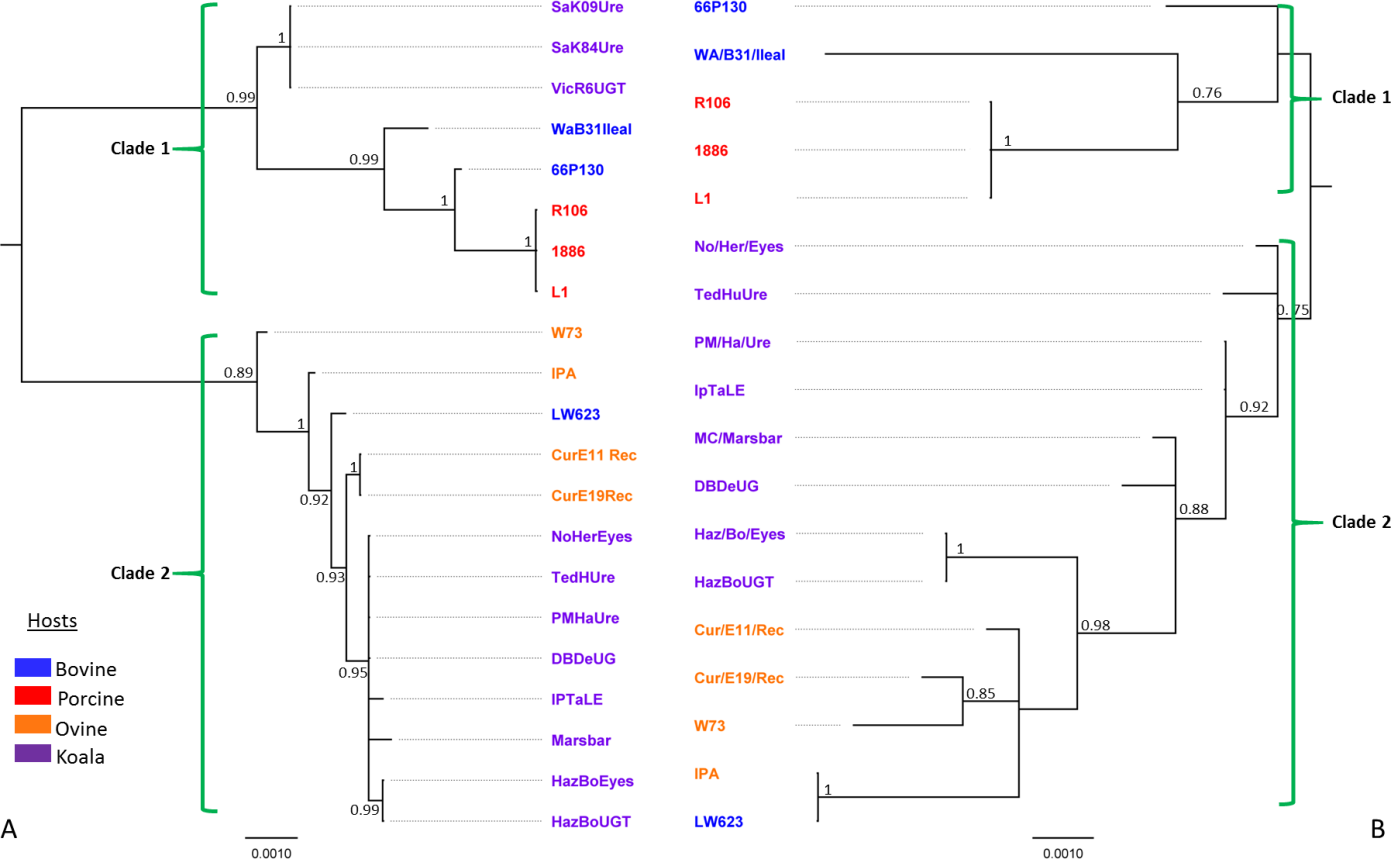
Bayesian phylogenetic analyses of (A) 7.5 kbp 21 p*Cpec* sequences from *C. pecorum* strains from porcine, ovine, bovine, and koala hosts; and (B) concatenated sequences of the seven MLST *C. pecorum* genes from 18 corresponding strains harbouring plasmids. Posterior probabilities >0.75 are displayed on the tree nodes, while the hosts are indicated by the colouring on the legend.

We also compared the phylogenies for our p*Cpec* sequences (Fig. 2A) to the corresponding MLST phylogenetic tree for the strains harbouring these plasmids (Fig. 2B). In the absence of whole genome sequences for a number of isolates, MLST-derived phylogenies were utilised, as they have previously been shown to be congruent with those constructed from core genome alignments (Bachmann et al., 2014; Bachmann et al., 2015). Using the *C. pneumoniae* plasmid sequence as an out-group, the root of the *C. pecorum* plasmid tree falls between two distinct p*Cpec* clades (Fig. S3). Further, the phylogenetic tree also resolved 21 p*Cpec* sequences into lineages according to their genotype and/or closely related genotypes (Figs. 2A and S2B). Clade 1 included a distinct lineage consisting of p*Cpec*s from South Australian (SA) and Victorian (Vic) koala strains of an identical genotype, as well as the more diverse lineage that consisted of the bovine p*Cpec*66P130 and p*Cpec*WAB31Ileal, and porcine p*Cpec*s L1, 1886, and R106 sequences (Fig. 2A). The genetically diverse Clade 2 included all the remaining eight p*Cpec*s from Queensland (QLD) and New South Wales (NSW) koala *C. pecorum* strains forming a well-supported lineage, as well as the p*Cpec*s CurE11Rec, CurE19Rec from Australian sheep strains, and p*Cpec*LW623, p*Cpec*W73 and p*Cpec*IPA from USA bovine and ovine *C. pecorum* isolates, respectively (Fig. 2A). p*Cpec* phylogeny was found to be largely congruent with a *C. pecorum* MLST phylogeny (Fig. 2B). In the MLST tree, we also observed two distinct clades. The first clade included lineages from porcine and bovine *C. pecorum* (Fig. 2B), while the second genetically diverse clade included all the koala *C. pecorum* strains resolved into their own lineages, and the four ovine (W73, CurE11Rec, CurE19Rec, and IPA) and the bovine LW623 MLST sequences (Fig. 2B).

Overall, this phylogenetic analysis revealed similar levels of inter-host *C. pecorum* strain genetic variability to that described previously using various other molecular markers (Jelocnik et al., 2015b; Mohamad et al., 2014), as well as congruency with our previously published *C. pecorum* core genome phylogenies (Bachmann et al., 2014; Jelocnik et al., 2015a). The co-evolution of plasmids with the chromosome of *C. pecorum* is consistent with that previously described for *C. trachomatis* (Seth-Smith et al., 2009). Separation of the p*Cpec* sequences into two distinct clades with different lineages suggests that, like the pathogen itself, the current *C. pecorum* plasmids are of a polyphyletic origin. Using p*Cpec*s from koala strains as an example, we observed an evolutionary split between the genetically identical plasmids from South Australian (SA) and Victorian (Vic) koala *C. pecorum* strains, and the more genetically diverse plasmids from Queensland (QLD) and New South Wales (NSW) koala strains. It should be also noted that the plasmids from QLD and NSW koala strains appear to be more closely related to the plasmids from sheep strains than to plasmids from strains from the same host, an observation that is consistent with our previous molecular typing and comparative genomics studies of these *C. pecorum* strains (Bachmann et al., 2015).

### Sequence analyses of pCpecs and its encoded proteins

Manually curated annotation of the 21 p*Cpec* sequences revealed a similar structure to that of chlamydial plasmids from other species with eight CDSs predicted in total. CDSs 1, 2, 3, 7 and 8 are putatively involved in plasmid maintenance and replication, while CDSs 4, 5 and 6 are associated with chlamydial-specific virulence (Gong et al., 2013; Song et al., 2013; Thomas et al., 1997) (Table 2). p*Cpec* also carry the four 22 bp tandem repeats located in the putative origin of replication (Figs. 1 and S2A).

**Table 2:** Characteristics of plasmid CDSs from 21 characterised p*Cpec* from *C. pecorum* pig, cattle, sheep, and koala strains.

Plasmid CDSs/ annotation	Predicted function	Length (bp)/ predicted a.a	No. of non- synonymous substitutions	d_n_^*^	No. of synonymous substitutions	d_s_^*^	*d_n_*∕*d_s_*	Δ*nt*	No. of alleles
CDS 1/***pGP8***	Integrase	936/312	2	0.00113	5	0.00995	0.113	7	4
CDS 2/***pGP8***	Integrase	1,026/342	6	0.00278	7	0.01280	0.217	13	8
CDS 3/***pGP1***	Replicative DNA helicase	1,374/458	4	0.00148	7	0.01130	0.131	11	6
CDS 4/***pGP2***	Virulence plasmid protein^a^	1,026/342	2	0.00074	10	0.01881	0.039	12	6
CDS 5/***pGP3***	Virulence plasmid protein^a^	795/265	4	0.00290	6	0.01219	0.131	10	6
CDS 6/***pGP4***	Virulence plasmid protein^a^	309/103	0	0	3	0.01654	0	3	4
CDS 7/***Par A***	Plasmid partitioning protein	783/261	2	0.00097	8	0.01838	0.053	10	4
CDS 8/***pGP6***	Plasmid replication protein	744/248	2	0.01839	7	0.00141	0.077	9	6
Intergenic region (between CDSs 8 and 1)	Origin of replication	207/4 × 22 bp tandem repeats	n.a	n.a	n.a	n.a	n.a	2	3

**Notes.**

a *Chlamydia* specific.

* *d_s_* and *d_n_*, the average number of synonymous substitutions per synonymous site and non-synonymous substitutions per non-synonymous site, respectively (Jukes—Cantor corrected); Δ*nt*, No. of polymorphic sites; No. of alleles, No. of unique sequences of each CDS.

The alignment of the 21 full length p*Cpec* sequences derived either from genome sequencing or PCR-based approaches, revealed from 0 to 83 SNPs (a maximum split between the two p*Cpec*s sequences) distributed evenly around the plasmid. A single insertion of 1 bp was seen in bovine 66P130 and LW623, and porcine L1, 1886 and R106 p*Cpec*s located in the intergenic region between CDS 6 and CDS 7 (Fig. S2A). Using the reconstructed plasmid sequence as a reference, we also identified p*Cpec* homoplasic SNPs. As observed in the p*Cpec* SNPs-only alignment (Fig. S2B), we detected 10 homoplasic SNPs, three of them (at positions 9, 10 and 84) in the intergenic regions, while the remaining seven (at positions 1, 16, 29, 69, 75, 76 and 78) were in the p*Cpec* CDSs 1, 2, 3, 7 and 8. Homoplasic SNPs at positions 1, 16, 76 and 78 also resulted in a non-synonymous amino acid change (Fig. S2B). Although the most parsimonious explanation is for putative recombination between these strains, in our present analyses using a set of 21 p*Cpec*s we do not have an evidence of recombination, hence it is uncertain whether the observed homoplasic SNPs occurred as a result of a homologous recombination or by independent selection (Harris et al., 2012).

We further assessed each of the eight CDSs as well as the intergenic regions for synonymous and non-synonymous SNPs (Table 2). Most SNPs were detected in the CDS 2 (putative integrase) and CDS 4 (putative plasmid virulence protein), with 13 and 12 SNPs each, respectively. p*Cpec*s CDS 2 also had the most non-synonymous changes (*n* = 6) (Fig. S2A) and could be detected in eight different alleles. In contrast to the seven polymorphic CDSs with seven to 13 SNPs each, p*Cpec*s CDS 6 was the most conserved sequence, with only three synonymous SNPs.

The level and distribution of the p*Cpec* sequence variation was comparable to that in *C. trachomatis* plasmids (Ferreira et al., 2013; Seth-Smith et al., 2009). Using only a limited set of p*Cpec* sequences, in our analyses the sequence variation mostly appears to be a result of synonymous changes. Nevertheless, it was interesting to note that most of the non-synonymous changes, including the ones in virulence-associated CDSs 4 and 5, seem to be accumulating in p*Cpec*s from *C. pecorum* strains derived from sheep, and QLD and NSW koalas presenting with disease (Fig. S2A). In regards to the plasmid virulence-associated CDSs, CDS 4 was the most polymorphic locus with only two non-synonymous changes, however. *C. pecorum* CDS 5 (*pgp*3) had the most non-synonymous changes (*n* = 4), mainly accumulating in the 5’end of the gene, while the CDS 6 was the most conserved. *C. trachomatis* CDS 5, encoding PgP3, is the most polymorphic CDS in this plasmid (Ferreira et al., 2013; Seth-Smith et al., 2009). Cytosol-exported PgP3 has been implicated as one of the major chlamydial factors affecting disease pathogenesis in *C. trachomatis* (Li et al., 2008), therefore possibly due to the host’s immune pressure, this CDS is under pressure to accumulate SNPs. At present, it is unknown what selective pressures are being placed on the *Chlamydia*-specific virulence-associated p*Cpec* CDS 4 and 5 genes. Future studies to investigate this will require a larger set of p*Cpec*s from strains isolated from a variety of diseased as well as healthy hosts, using both bioinformatics as well as cell biology approaches.

Detection of the most variation and non-synonymous changes in p*Cpec* CDS 2 contrasts with its strong conservation in the *C. trachomatis* plasmid. The remaining p*Cpec* CDSs displayed high sequence conservation, particularly for CDS 7 and CDS 8. These CDSs are putatively denoted as the partitioning co-transcribing genes (Ferreira et al., 2013), and their encoded proteins may play essential role in effective plasmid segregation to the progeny cells, hence their sequence conservation is not surprising. Based on our findings, p*Cpec* sequences appear to be evolutionary conserved and it is likely that the function of the encoded proteins is similar to that previously predicted for *C. trachomatis* (Seth-Smith et al., 2009), and *C. pneumoniae* (Thomas et al., 1997).

## Conclusions

In the present study, we have characterised the genetic structure and phylogenetic relationships of 21 p*Cpec* from porcine, ovine, bovine, and koala strains. Our data suggests that the p*Cpec* sequence is evolutionarily conserved, as it is in related chlamydial species, with the chlamydial plasmid virulence-associated features present. The p*Cpec*phylogenies revealed “co-evolution” of plasmids with their respective *C. pecorum* chromosomes, further supporting a polyphyletic evolution of this pathogen at least in Australian koalas (Fig. 2). Based on the p*Cpec* sequence and predicted gene functions, the level and nature of the plasmid conservation suggests that p*Cpec*, where found, is potentially important for during growth, infection, and/or transmission of the bacterium within a population, as has been suggested in studies comparing plasmid-positive and plasmid-negative chlamydial isolates (Rockey, 2011; Russell et al., 2011). Although how this relates to the plasmid-negative *C. pecorum* is not clear at present. This study provides more clues to understand the complex epidemiology of this pathogen in livestock and koala hosts.

## Supplemental Information

10.7717/peerj.1661/supp-1 Figure S1 Bayesian phylogenetic analyses of plasmid sequences from eight related chlamydial species, compared to the 16S rRNA gene sequences from corresponding chlamydial strains harbouring these plasmids Bayesian phylogenetic analyses of (A) plasmid sequences from eight related chlamydial species, compared to the (B) 16S rRNA gene sequences from corresponding chlamydial strains harbouring these plasmids. Posterior probabilities >0.75 are displayed on the tree nodes. C. muridarum sequences were used as an out-group. Associated plasmid and 16S rRNA gene sequence from the same chlamydial strain are denoted by coloured arrows.

10.7717/peerj.1661/supp-2 Figure S2 pCpec SNP distribution and SNP phylogeny (A) SNP distribution in the p*Cpec* genotypes, using Genotype A as a reference. SNP positions are highlighted in black, while the type of variants are highlighted in purple for A, pink for G, green for C, light blue for T. SNPs resulting in non-synonymous changes are indicated with red boxes. A single bp insertion in the p*Cpec* genotypes I, J and L are indicated with a green box. The 22bp tandem repeat units are indicated by blue arrows. (B) The p*Cpec* phylogeny aligned to the tracks of p*Cpec* SNP only alignment, using reconstructed plasmid sequence N1 as a reference. Above the alignment is the graphical representation of p*Cpec* CDSs position in reference to the SNPs alignment, while the top line is numbering the successive SNPs as detected in the p*Cpec* sequences. SNPs are highlighted as disagreements to the reference sequence. Homoplasic SNPs are denoted with star symbols. Ones resulting in a non-synonymous change are denoted with red stars, while the ones resulting in a synonymous change are denoted with blue stars.

10.7717/peerj.1661/supp-3 Figure S3 Neighbor-Joining phylogenetic analyses of the 21 p*Cpec* sequences from *C. pecorum* strains from porcine, ovine, bovine, and koala hosts using *C. pneumoniae* pLPCoLN as an out-group Neighbor-Joining phylogenetic analyses of the 21 p*Cpec* sequences from *C. pecorum* strains from porcine, ovine, bovine, and koala hosts using *C. pneumoniae* pLPCoLN as an out-group. Bootstrap values (1,000 times repetitions) are displayed on the tree nodes.

10.7717/peerj.1661/supp-4 Table S1 Primers used for generating plasmid (pCpec) fragments and their characteristics

## Additional Information and Declarations

### Data Availability

The following information was supplied regarding data availability: Plasmid sequences were deposited in Genbank under accession numbers KT223766– KT223781, and KT352920, KT352921, KT352922, KT352923and KT352924.

## References

[ref-1] Andersson SGE, Kurland CG (1998). Reductive evolution of resident genomes. Trends in Microbiology.

[ref-2] Ashkenazy H, Penn O, Doron-Faigenboim A, Cohen O, Cannarozzi G, Zomer O, Pupko T (2012). FastML: a web server for probabilistic reconstruction of ancestral sequences. Nucleic Acids Research.

[ref-3] Aziz RK, Bartels D, Best AA, DeJongh M, Disz T, Edwards RA, Formsma K, Gerdes S, Glass EM, Kubal M, Meyer F, Olsen GJ, Olson R, Osterman AL, Overbeek RA, McNeil LK, Paarmann D, Paczian T, Parrello B, Pusch GD, Reich C, Stevens R, Vassieva O, Vonstein V, Wilke A, Zagnitko O (2008). The RAST Server: rapid annotations using subsystems technology. BMC Genomics.

[ref-4] Bachmann N, Fraser T, Bertelli C, Jelocnik M, Gillett A, Funnell O, Flanagan C, Myers GS, Timms P, Polkinghorne A (2014). Comparative genomics of koala, cattle and sheep strains of *Chlamydia pecorum*. BMC Genomics.

[ref-5] Bachmann NL, Sullivan MJ, Jelocnik M, Myers GSA, Timms P, Polkinghorne A (2015). Culture-independent genome sequencing of clinical samples reveals an unexpected heterogeneity of infections by *Chlamydia pecorum*. Journal of Clinical Microbiology.

[ref-6] Chen J, Yang Z, Sun X, Tang L, Ding Y, Xue M, Zhou Z, Baseman J, Zhong G (2015). Intrauterine Infection with plasmid-free *Chlamydia muridarum* reveals a critical role of the plasmid in chlamydial ascension and establishes a model for evaluating plasmid-independent pathogenicity. Infection and Immunity.

[ref-7] Darriba D, Taboada GL, Doallo R, Posada D (2012). jModelTest 2: more models, new heuristics and parallel computing. Nature Methods.

[ref-8] Donati M, Laroucau K, Storni E, Mazzeo C, Magnino S, Di Francesco A, Baldelli R, Ceglie L, Renzi M, Cevenini R (2009). Serological response to pgp3 protein in animal and human chlamydial infections. Veterinary Microbiology.

[ref-9] Dugan J, Rockey DD, Jones L, Andersen AA (2004). Tetracycline resistance in *Chlamydia suis* mediated by genomic islands inserted into the chlamydial *inv*-like gene. Antimicrobial Agents and Chemotherapy.

[ref-10] Ferreira R, Borges V, Nunes A, Borrego MJ, Gomes JP (2013). Assessment of the load and transcriptional dynamics of *Chlamydia trachomatis* plasmid according to strains’ tissue tropism. Microbiological Research.

[ref-11] Gong S, Yang Z, Lei L, Shen L, Zhong G (2013). Characterization of *Chlamydia trachomatis* plasmid-encoded open reading frames. Journal of Bacteriology.

[ref-12] Gupta R, Naushad S, Chokshi C, Griffiths E, Adeolu M (2015). A phylogenomic and molecular markers based analysis of the phylum *Chlamydiae*: proposal to divide the class *Chlamydiia* into two orders, *Chlamydiales* and *Parachlamydiales* ord. nov., and emended description of the class *Chlamydiia*. Antonie van Leeuwenhoek.

[ref-13] Harris SR, Clarke IN, Seth-Smith HMB, Solomon AW, Cutcliffe LT, Marsh P, Skilton RJ, Holland MJ, Mabey D, Peeling RW, Lewis DA, Spratt BG, Unemo M, Persson K, Bjartling C, Brunham R, de Vries HJC, Morre SA, Speksnijder A, Bebear CM, Clerc M, de Barbeyrac B, Parkhill J, Thomson NR (2012). Whole-genome analysis of diverse *Chlamydia trachomatis* strains identifies phylogenetic relationships masked by current clinical typing. Nature Genetics.

[ref-14] Jelocnik M, Bachmann NL, Kaltenboeck B, Waugh C, Woolford L, Speight K Natasha, Gillett A, Higgins DP, Flanagan C, Myers Garry SA, Timms P, Polkinghorne A (2015a). Genetic diversity in the plasticity zone and the presence of the chlamydial plasmid differentiates *Chlamydia pecorum* strains from pigs, sheep, cattle, and koalas. BMC Genomics.

[ref-15] Jelocnik M, Forshaw D, Cotter J, Roberts D, Timms P, Polkinghorne A (2014a). Molecular and pathological insights into *Chlamydia pecorum*-associated sporadic bovine encephalomyelitis (SBE) in Western Australia. BMC Veterinary Research.

[ref-16] Jelocnik M, Frentiu FD, Timms P, Polkinghorne A (2013). Multi-locus sequence analysis provides insights into the molecular epidemiology of *Chlamydia pecorum* infections in Australian sheep, cattle and koalas. Journal of Clinical Microbiology.

[ref-17] Jelocnik M, Self R, Timms P, Borel N, Polkinghorne A (2015b). Novel sequence types of *Chlamydia pecorum* infect free-ranging Alpine ibex (*Capra ibex*) and red deer (*Cervus elaphus*) in Switzerland. Journal of Wildlife Diseases.

[ref-18] Jelocnik M, Walker E, Pannekoek Y, Ellem J, Timms P, Polkinghorne A (2014b). Evaluation of the relationship between *Chlamydia pecorum* sequence types and disease using a species-specific multi-locus sequence typing scheme (MLST). Veterinary Microbiology.

[ref-19] Kaltenboeck B, Kousoulas KG, Storz J (1993). Structures of and allelic diversity and relationships among the major outer membrane protein (*omp*A) genes of the four chlamydial species. Journal of Bacteriology.

[ref-20] Kari L, Whitmire WM, Olivares-Zavaleta N, Goheen MM, Taylor LD, Carlson JH, Sturdevant GL, Lu C, Bakios LE, Randall LB, Parnell MJ, Zhong G, Caldwell HD (2011). A live-attenuated chlamydial vaccine protects against trachoma in nonhuman primates. The Journal of Experimental Medicine.

[ref-21] Kearse M, Moir R, Wilson A, Stones-Havas S, Cheung M, Sturrock S, Buxton S, Cooper A, Markowitz S, Duran C, Thierer T, Ashton B, Meintjes P, Drummond A (2012). Geneious Basic: an integrated and extendable desktop software platform for the organization and analysis of sequence data. Bioinformatics.

[ref-22] Koelbl O (1969). Untersuchungen ueber das vorkommen von *Miyagawanellen* beim schwein. Wien Tierarztl Mschr.

[ref-23] Li Z, Chen D, Zhong Y, Wang S, Zhong G (2008). The chlamydial plasmid-encoded protein pgp3 is secreted into the cytosol of *Chlamydia*-infected cells. Infection and Immunity.

[ref-24] Librado P, Rozas J (2009). DnaSP v5: a software for comprehensive analysis of DNA polymorphism data. Bioinformatics.

[ref-25] Liu Y, Huang Y, Yang Z, Sun Y, Gong S, Hou S, Chen C, Li Z, Liu Q, Wu Y, Baseman J, Zhong G (2014). Plasmid-encoded Pgp3 is a major virulence factor for *Chlamydia muridarum* to induce hydrosalpinx in mice. Infection and Immunity.

[ref-26] Mitchell C, Hovis K, Bavoil P, Myers G, Carrasco J, Timms P (2010). Comparison of koala LPCoLN and human strains of *Chlamydia pneumoniae* highlights extended genetic diversity in the species. BMC Genomics.

[ref-27] Mohamad KY, Kaltenboeck B, Rahman KhS, Magnino S, Sachse K, Rodolakis A (2014). Host adaptation of *Chlamydia pecorum* towards low virulence evident in co-evolution of the *omp*A, *inc*A, and ORF663 Loci. PLoS ONE.

[ref-28] Mojica S, Huot Creasy H, Daugherty S, Read TD, Kim T, Kaltenboeck B, Bavoil P, Myers GSA (2011). Genome sequence of the obligate intracellular animal pathogen *Chlamydia pecorum* E58. Journal of Bacteriology.

[ref-29] Nunes A, Gomes JP (2014). Evolution, phylogeny, and molecular epidemiology of *Chlamydia*. Infection, Genetics and Evolution.

[ref-30] Peterson EM, Markoff BA, Schachter J, de la Maza LM (1990). The 7.5-kb plasmid present in *Chlamydia trachomatis* is not essential for the growth of this microorganism. Plasmid.

[ref-31] Pickett MA, Everson JS, Pead PJ, Clarke IN (2005). The plasmids of *Chlamydia trachomatis* and *Chlamydophila pneumoniae* (N16): accurate determination of copy number and the paradoxical effect of plasmid-curing agents. Microbiology.

[ref-32] Read TD, Joseph SJ, Didelot X, Liang B, Patel L, Dean D (2013). Comparative analysis of *Chlamydia psittaci* genomes reveals the recent emergence of a pathogenic lineage with a broad host range. MBio.

[ref-33] Ripa T, Nilsson PA (2007). A *Chlamydia trachomatis* strain with a 377-bp deletion in the cryptic plasmid causing false-negative nucleic acid amplification tests. Sexually Transmitted Diseases.

[ref-34] Rockey DD (2011). Unraveling the basic biology and clinical significance of the chlamydial plasmid. Journal of Experimental Medicine.

[ref-35] Russell M, Darville T, Chandra-Kuntal K, Smith B, Andrews CW, O’Connell CM (2011). Infectivity acts as *in vivo* selection for maintenance of the chlamydial cryptic plasmid. Infection and Immunity.

[ref-36] Sait M, Clark EM, Wheelhouse N, Livingstone M, Spalding L, Siarkou VI, Vretou E, Smith DGE, Lainson FA, Longbottom D (2011). Genome sequence of the *Chlamydophila abortus* variant strain LLG. Journal of Bacteriology.

[ref-37] Sait M, Livingstone M, Clark EM, Wheelhouse N, Spalding L, Markey B, Magnino S, Lainson FA, Myers GS, Longbottom D (2014). Genome sequencing and comparative analysis of three *Chlamydia pecorum* strains associated with different pathogenic outcomes. BMC Genomics.

[ref-38] Seth-Smith HM, Harris SR, Persson K, Marsh P, Barron A, Bignell A, Bjartling C, Clark L, Cutcliffe LT, Lambden PR, Lennard N, Lockey SJ, Quail MA, Salim O, Skilton RJ, Wang Y, Holland MJ, Parkhill J, Thomson NR, Clarke IN (2009). Co-evolution of genomes and plasmids within *Chlamydia trachomatis* and the emergence in Sweden of a new variant strain. BMC Genomics.

[ref-39] Sigar IM, Schripsema JH, Wang Y, Clarke IN, Cutcliffe LT, Seth-Smith HM, Thomson NR, Bjartling C, Unemo M, Persson K, Ramsey KH (2014). Plasmid deficiency in urogenital isolates of *Chlamydia trachomatis* reduces infectivity and virulence in a mouse model. Pathogens and Disease.

[ref-40] Song L, Carlson JH, Whitmire WM, Kari L, Virtaneva K, Sturdevant DE, Watkins H, Zhou B, Sturdevant GL, Porcella SF, McClarty G, Caldwell HD (2013). *Chlamydia trachomatis* plasmid-encoded *Pgp*4 is a transcriptional regulator of virulence-associated genes. Infection and Immunity.

[ref-41] Stothard DR, Williams JA, Van Der Pol B, Jones RB (1998). Identification of a *Chlamydia trachomatis* serovar E urogenital isolate which lacks the cryptic plasmid. Infection and Immunity.

[ref-42] Thomas NS, Lusher M, Storey CC, Clarke IN (1997). Plasmid diversity in *Chlamydia*. Microbiology.

