# Supplementary figures and images for: Molecular characterisation of the Chlamydia pecorum plasmid from porcine, ovine, bovine, and koala strains indicates plasmid-strain co-evolution
Source: PeerJ. 2016 Feb 4;4:e1661. doi: 10.7717/peerj.1661 (PMC4748734; doi:10.7717/peerj.1661)

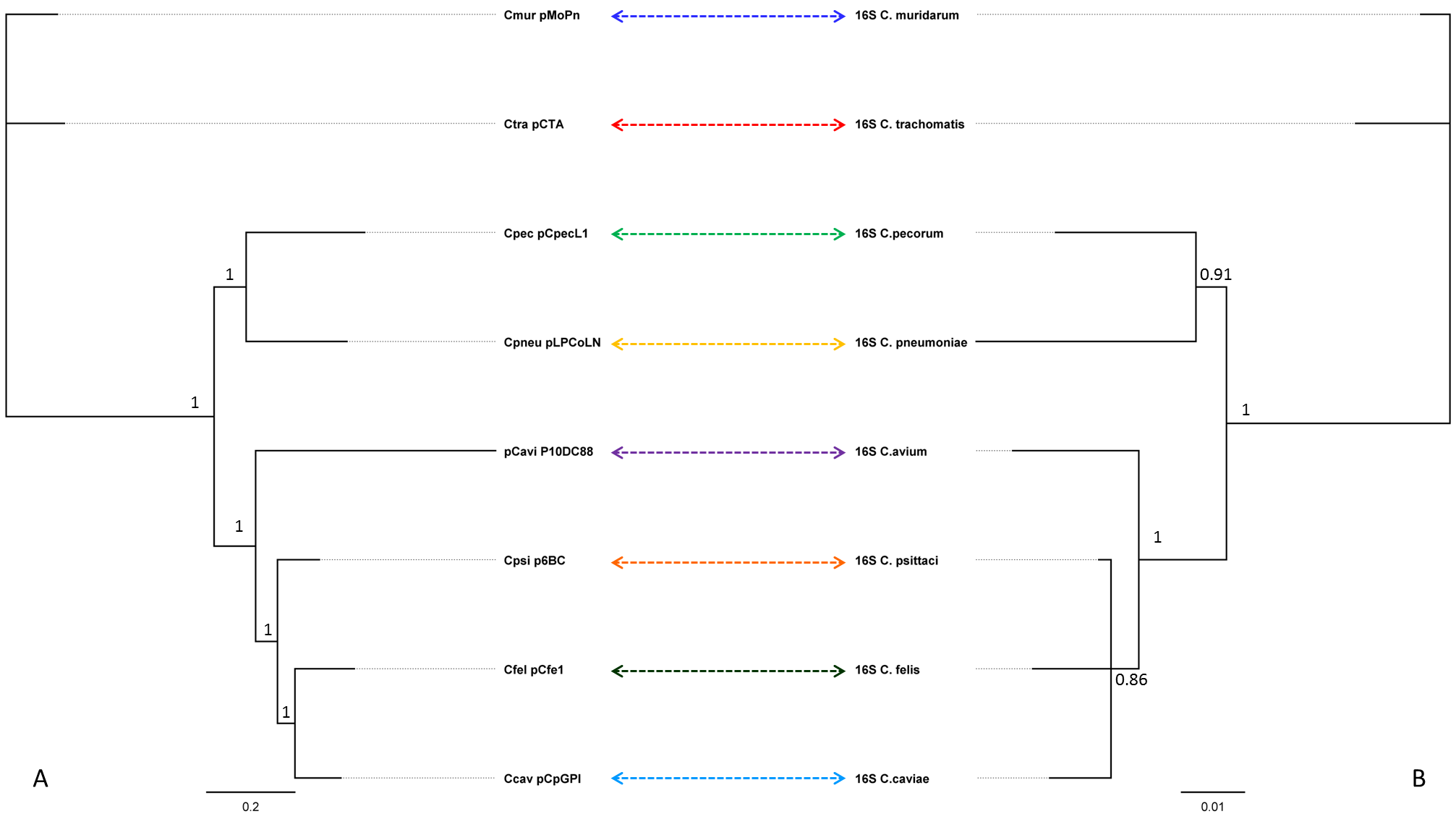

Supplement: Figure S1 — Bayesian phylogenetic analyses of (A) plasmid sequences from eight related chlamydial species, compared to the (B) 16S rRNA gene sequences from corresponding chlamydial strains harbouring these plasmids. Posterior probabilities >0.75 are displayed on the tree nodes. C. muridarum sequences were used as an out-group. Associated plasmid and 16S rRNA gene sequence from the same chlamydial strain are denoted by coloured arrows. [file peerj-04-1661-s001.png]

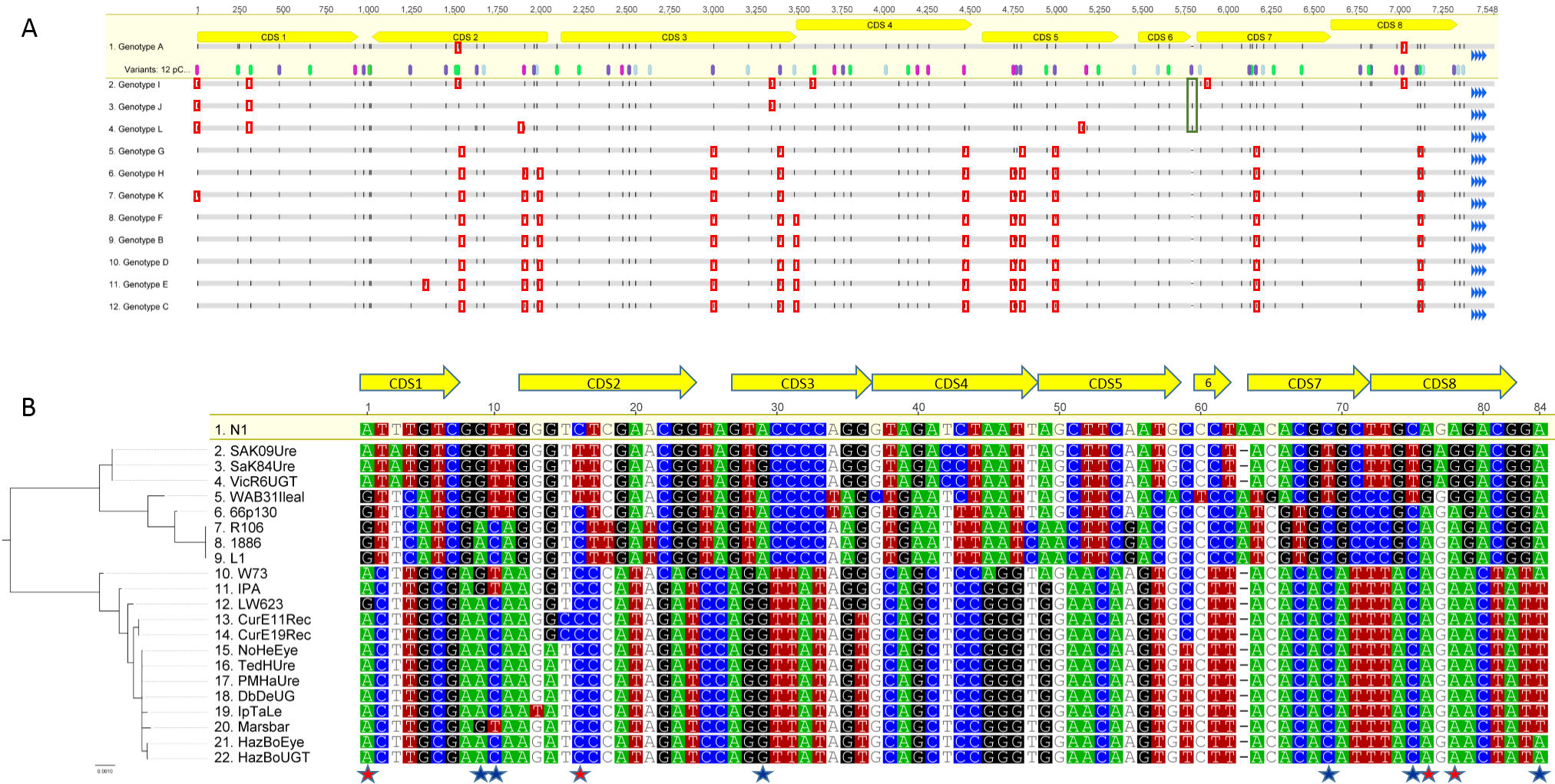

Supplement: Figure S2 — (A) SNP distribution in the pCpec genotypes, using Genotype A as a reference. SNP positions are highlighted in black, while the type of variants are highlighted in purple for A, pink for G, green for C, light blue for T. SNPs resulting in non-synonymous changes are indicated with red boxes. A single bp insertion in the pCpec genotypes I, J and L are indicated with a green box. The 22bp tandem repeat units are indicated by blue arrows. (B) The pCpec phylogeny aligned to the tracks of pCpec SNP only alignment, using reconstructed plasmid sequence N1 as a reference. Above the alignment is the graphical representation of pCpec CDSs position in reference to the SNPs alignment, while the top line is numbering the successive SNPs as detected in the pCpec sequences. SNPs are highlighted as disagreements to the reference sequence. Homoplasic SNPs are denoted with star symbols. Ones resulting in a non-synonymous change are denoted with red stars, while the ones resulting in a synonymous change are denoted with blue stars. [file peerj-04-1661-s002.png]

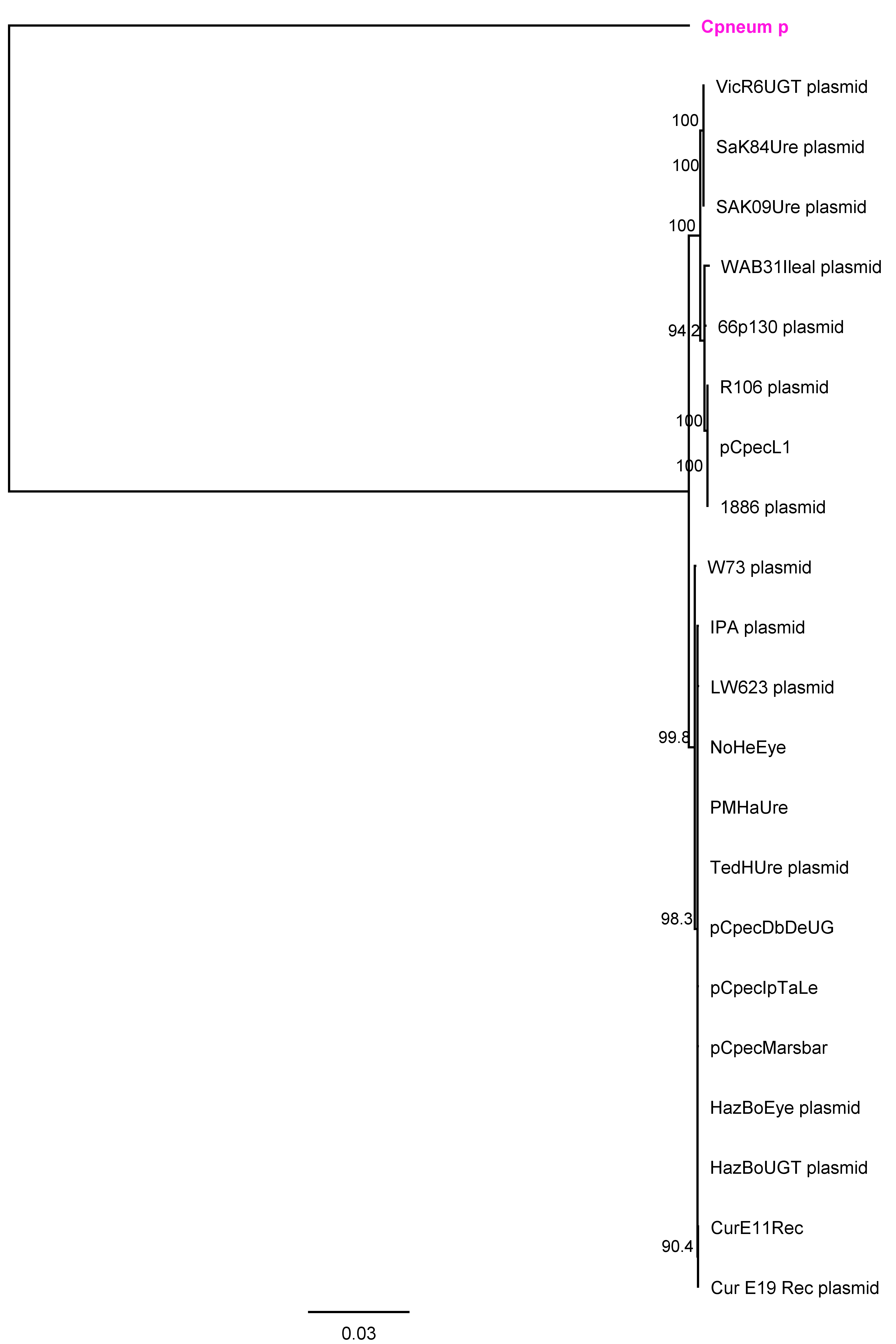

Supplement: Figure S3 — Neighbor-Joining phylogenetic analyses of the 21 pCpec sequences from C. pecorum strains from porcine, ovine, bovine, and koala hosts using C. pneumoniae pLPCoLN as an out-group. Bootstrap values (1,000 times repetitions) are displayed on the tree nodes. [file peerj-04-1661-s003.png]
