# Supplementary material for: Molecular characterisation of the Chlamydia pecorum plasmid from porcine, ovine, bovine, and koala strains indicates plasmid-strain co-evolution
Source: PeerJ. 2016 Feb 4;4:e1661. doi: 10.7717/peerj.1661 (PMC4748734; doi:10.7717/peerj.1661)
Supplement: Table S1 [file peerj-04-1661-s004.pdf]

Table S1. Primers used for generating plasmid (p*Cpec*) fragments and their characteristics.

Primer name	Sequence 5' - 3'	Primer length (bp)	Annealing temperature ($Tm^{o}C$)	Region on the plasmid^	Amplified fragment (bp)
**Start For**	TAG CTC GTA ATA CGT GTG G	19	56	775	792
**Start Rev**	ATG AGG CAG AGT GTG AAC C	19	58	1	
**P1 frag For**	GTT CAC ACT CTG CCT CAT C	19	60	7046	522
**P frag Rev**	CCT ATT TAT TGG CGT CTA GG	20	58	19	
**PG6 For**	GCA GAA TTG GGT AGT CCT TG	20	60	7127	802
**Par Rev**	TCA ATC TTT AGA AGT TTT GGG	21	56	6325	
**Par For**	CTT CTA AAG ATT GAT CTA GGT	21	56	6338	702
**PG4 Rev**	ATA CTG AAC CTC CTA AAT CG	20	56	5636	
**PG4 For**	ATC TAT AAT CGG AGA ATC TGG	21	58	5701	836
**PG3 Rev**	AGA ATT GAT AGA AGA TGT GC	20	54	4865	
**PG3 For**	GTT CCC ATT TGG CAT TGC GT	20	60	5297	1213
**PG2 Rev**	TTG AAC CAT GCC CTA TTA TCG	21	60	4084	
**PG2 For**	GCT GTG CTG ACA ATC CAG T	19	58	4225	918
**Hel Rev**	GGT CAA ATA GAA CAA GAT GCT	21	56	3307	
**Hel For**	AGC ATC TTG TTC TAT TTG ACC	21	54	3327	840
**Hel 2 Rev**	CAA AGA AAT TCA TAG GTA TCC	21	56	2487	
**Hel 2 For**	TGA TCT ACA AGA GTA TAT GG	20	54	2540	840
**PG8 Rev**	AGA GAC TAT CTT CTG TGG G	19	56	1700	
**PG8 For**	TCA CTA AAT ACA CAT ACA GCC	21	54	1856	739
**P8 2 Rev**	GAG TCC GAA CAT CCA AGA CG	20	62	1119	
**P8 2 For**	CGA TTG GTT AAT AGC ACA GAC	21	60	1481	749
**End Rev**	ATT GGC AGA ACA TGA TGC TC	20	58	735	
**PGP3 Rev**	TTA ATC ATG GGT AAT TCA GG	20	54	4566	In combination with others

^: 1 at putative ORI (See plasmid map, Figure 1).
